# Rare, Locally Advanced Presentation of Giant Scalp Squamous Cell Carcinoma: A Case Report

**DOI:** 10.7759/cureus.110254

**Published:** 2026-06-04

**Authors:** Soufyane Elkadiri, Abdelhafid El Mrahi, Anas Ankiz, Leila Benahmed, Ismail Hailouma, Doha Arreyouchi, Zerrouki Nassiba, Ayat Allah Oufkir, Nada Zizi

**Affiliations:** 1 Department of Plastic and Reconstructive Surgery, Mohammed VI University Hospital Centre, Oujda, MAR; 2 Department of Dermatology, Venereology and Allergology, Mohammed VI University Hospital Centre, Oujda, MAR; 3 Department of Epidemiology, Clinical Research and Public Health, Mohamed I University, Oujda, MAR; 4 Department of Dermatology, Venereology and Allergology, Mohammed VI University Hospital, Oujda, MAR

**Keywords:** giant cutaneous squamous cell carcinoma, multidisciplinary management, scalp reconstruction, scalp tumor, surgical excision

## Abstract

Giant cutaneous squamous cell carcinoma (cSCC) of the scalp is rare and presents significant therapeutic challenges due to its size, proximity to critical neurovascular structures, and complex reconstructive requirements. We report a 68-year-old woman with a 20 cm exophytic occipito-parietal cSCC that had been evolving over one year. Biopsy confirmed a well-differentiated cSCC, and imaging revealed a heterogeneous mass abutting the skull without nodal or distant metastases. The tumor was managed with wide surgical excision, including the cranial periosteum and outer table curettage, followed by staged reconstruction using pedicled transposition flaps and split-thickness skin grafts. Histopathology confirmed a well-differentiated infiltrative cSCC (T3N0M0, American Joint Committee on Cancer (AJCC) stage III) with clear margins. Postoperative outcomes were satisfactory, both functionally and aesthetically. This case underscores the importance of early detection, multidisciplinary management, and individualized surgical planning in giant scalp cSCC, with adjuvant therapies offering additional options for advanced disease.

## Introduction

Cutaneous squamous cell carcinoma (cSCC) is the second most common skin cancer after basal cell carcinoma, accounting for approximately 20% of non-melanoma skin cancers. Its incidence continues to rise worldwide, particularly among elderly individuals with chronic ultraviolet (UV) exposure and immunocompromised patients. The diagnosis is based on both clinical evaluation and histopathological analysis. Because these tumors may resemble benign cutaneous lesions, tissue biopsy remains indispensable for establishing a definitive diagnosis [1].

According to the American Joint Committee on Cancer (AJCC) staging system, tumor size, depth of invasion, and high-risk pathological features are key prognostic determinants that guide treatment decisions and outcomes. While most cSCCs are diagnosed at an early stage with an excellent prognosis, advanced tumors are associated with worse outcomes, including local recurrence, deep tissue invasion, and metastasis [2]. In recent years, systemic therapies, particularly immune checkpoint inhibitors such as anti-programmed cell death protein 1 (PD-1) agents, have emerged as an important option in the management of unresectable or metastatic cSCC, complementing surgery and radiotherapy in selected cases [3].

Giant cSCCs, commonly defined as lesions exceeding 5 cm in diameter, represent a rare and aggressive variant with poorer prognostic implications compared with conventional tumors. Their occurrence in the scalp is particularly uncommon and poses significant therapeutic challenges due to potential calvarial invasion, proximity to neurovascular structures, and complex reconstructive requirements following wide excision [4-6].

We report an exceptional case of a giant occipitoparietal cSCC measuring 20 cm in a 68-year-old woman, highlighting the diagnostic and therapeutic challenges associated with rapidly progressive advanced scalp cSCC and emphasizing the importance of early recognition and multidisciplinary management.

## Case presentation

A 68-year-old woman with Fitzpatrick skin type III [7] and a history of chronic sun exposure, ischemic cardiomyopathy with reduced left ventricular systolic function (LVEF 35%), and no prior surgical or family history, presented with a painless exophytic scalp mass evolving for one year. The lesion was initially nodular and increased rapidly in size despite self-medication, impairing her quality of life.

Clinical examination revealed a large, polylobulated, cauliflower-like occipito-parietal mass measuring 20 cm. The lesion was painless, fixed, and covered with hemorrhagic and honey-colored crusts. Regional lymph node examination was unremarkable, and the remainder of the skin examination was normal. Clinically, the tumor was highly suggestive of malignancy, raising the differential diagnoses of basal cell carcinoma, squamous cell carcinoma, sarcoma, or angiosarcoma (Figure 1).

**Figure 1:**
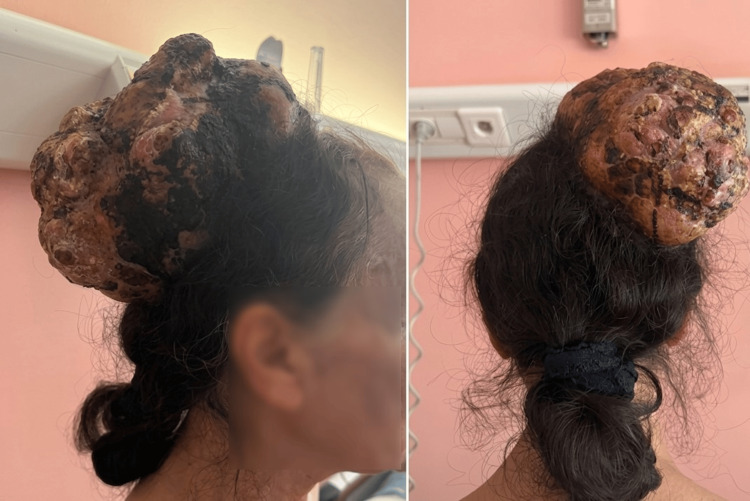
Polylobulated, cauliflower-like occipito-parietal mass

An initial biopsy confirmed squamous cell carcinoma (SCC). Brain CT demonstrated a right parieto-occipital mass measuring 167 × 190 × 80 mm, polylobulated and heterogeneous, containing cystic and hemorrhagic components, and in close contact with the outer table of the skull (Figure 2).

**Figure 2:**
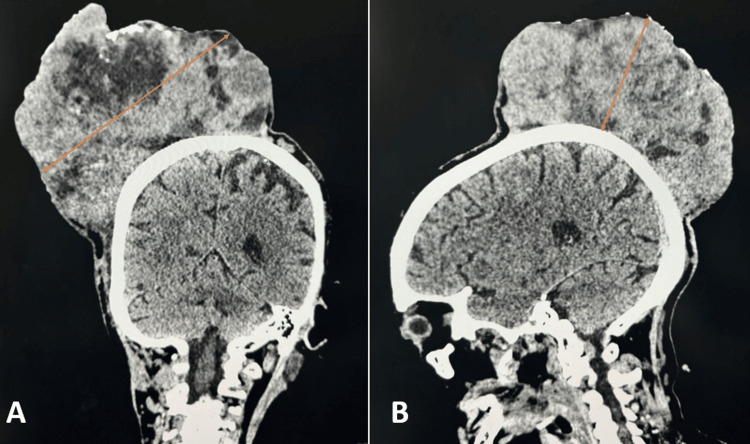
Contrast-enhanced CT scan A: coronal and B: sagittal, showing a heterogeneous exophytic right parieto-occipital scalp mass in contact with the outer skull table.

Cervico-thoraco-abdomino-pelvic computed tomography showed no evidence of regional lymph node involvement or distant metastases Laboratory investigations showed corrected hypercalcemia, and normal renal function.

Treatment consisted of wide surgical excision of the tumor with 1 cm safety margins, including resection of the cranial periosteum in depth. Additional deep resection was achieved by curettage of the outer table of the cranial bone, resulting in a substantial defect that was initially covered with paraffin gauze dressings. Scalp reconstruction was performed secondarily, after definitive histopathological confirmation of negative margins. Reconstruction consisted of three pedicled transposition flaps, combined with split-thickness grafts (Figure 3).

**Figure 3:**
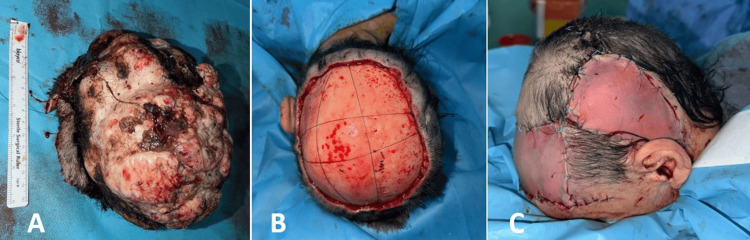
Intraoperative images (A) excised 20 cm tumor; (B) post-excision defect; and (C) scalp reconstruction using three pedicled flaps with skin graft.

Histopathological examination confirmed a well-differentiated, infiltrative, keratinizing SCC. The tumor was staged as T3N0M0, corresponding to stage III according to the AJCC 8th edition classification [2]. Surgical margins and bone resection were clear. No evidence of perineural invasion or lymphovascular invasion was identified on histopathological analysis (Figure 4).

**Figure 4:**
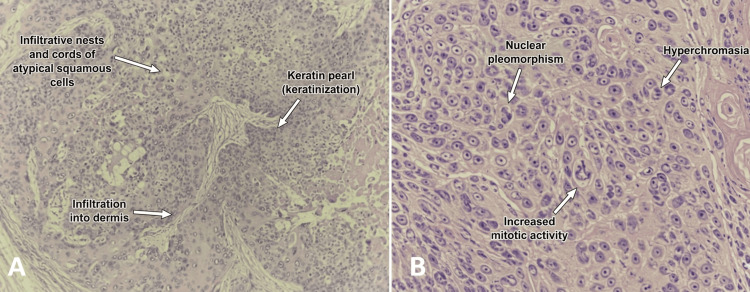
Hematoxylin and eosin (H&E) sections (A, B) show infiltrative squamous cell carcinoma with atypical squamous cells in nests and cords, marked pleomorphism, hyperchromasia, mitoses, and foci of keratinization with keratin pearl formation.

The postoperative course was favorable, with viable flaps and good graft take. Functional and aesthetic outcomes were considered satisfactory by the patient (Figure 5).

**Figure 5:**
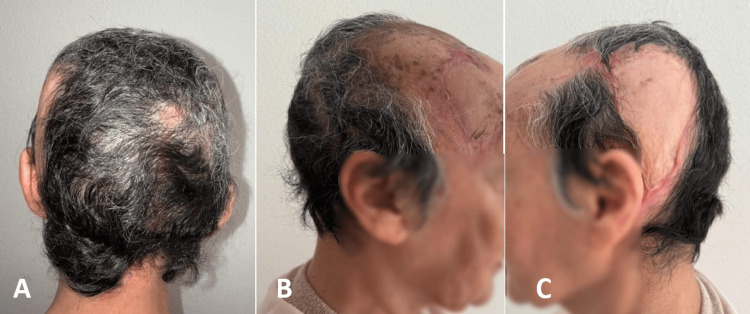
Postoperative appearance at the three-month follow-up showing viable flaps and good graft take

Adjuvant radiotherapy is planned after complete healing, as decided by the multidisciplinary tumor board. A structured follow-up protocol was established: every three months for the first two years, every six months for the subsequent three years, and every six months thereafter for life.

## Discussion

cSCC is the second most common non-melanoma skin cancer. Advanced age, fair phototype, prolonged sun exposure, chronic immunosuppression, and a history of skin cancer are major risk factors [8]. Most cSCCs are diagnosed at an early stage and treated effectively; however, a subset may progress rapidly and reach exceptional dimensions when neglected. Giant cSCCs, defined as tumors exceeding 5 cm in diameter [8], are rare, particularly outside the anogenital region, and pose major therapeutic challenges. These challenges are significant when the tumor is located on the scalp, owing to the proximity of critical neurovascular structures and the complexity of reconstructive procedures [1,4-6,9].

Most reported series involve elderly patients, frequently over 75 years of age [9], making our 68-year-old patient slightly younger than the average for this type of presentation. The 20 cm lesion observed in our case illustrates its exceptional nature, both in terms of size and location, and highlights the considerable growth potential of these tumors in the absence of early management.

The literature indicates that giant cSCCs account for less than 1% of non-melanoma skin cancers, and reported extra-anogenital cases remain limited, often described in small series of fewer than fifty patients [6]. Tumor size, depth of invasion, and histological infiltration are major risk factors for recurrence and metastasis [10]. In our case, despite the remarkable size and parieto-occipital extension, the tumor was well differentiated, with no bone involvement or distant metastases, allowing complete surgical excision and effective reconstruction. Local flaps were preferred over free flaps due to the patient’s advanced age and underlying ischemic cardiomyopathy, as this approach minimized operative time while preserving the hair-bearing scalp and improving aesthetic outcomes.

Given the tumor's large size and extensive local extension, adjuvant radiotherapy was recommended to reduce the risk of local recurrence [11]. This multidisciplinary approach, involving dermatology, plastic surgery, and onclogy, ensured optimal tumor control and functional outcomes.

For advanced or unresectable cSCCs, novel targeted therapies have emerged as promising treatment options. PD-1 inhibitors, such as cemiplimab, particularly when combined with radiotherapy, have demonstrated encouraging clinical outcomes [8,12,13], including giant or recurrent cSCCs, sometimes achieving substantial tumor regression and durable disease control. These immunotherapeutic options, underscore the value of a multimodal strategy for large tumors or those at high risk of recurrence [14]. No immunotherapy was administered due to its unavailability in our institution, as well as limited accessibility and high cost.

Our case highlights the rarity and exceptional presentation of a 20 cm giant scalp cSCC; the importance of early detection and monitoring of chronic cutaneous lesions; and the necessity of tailored multidisciplinary management [15].

## Conclusions

This case highlights the rarity and clinical complexity of extra-anogenital giant cSCC of the scalp. The rapid progression, exceptional tumor size, and chronic inflammatory background emphasize that any long-standing or atypical cutaneous lesion should prompt early biopsy and definitive diagnosis without delay. This report further underscores the critical importance of timely multidisciplinary management in guiding both oncologic and reconstructive strategies. Finally, it reflects the ongoing evolution of therapeutic options for advanced cutaneous malignancies, in which systemic treatments such as anti-PD-1 immunotherapy are increasingly incorporated into the management of unresectable or high-risk disease.

## References

[1] Leal Z, Hobbs M, Gill R, Banfield M, Akhdar M, Casadesus D (2024). Rapidly growing giant squamous cell carcinoma of the head: a case report. Cureus.

[2] Amin MB, Greene FL, Edge SB (2017). The eighth edition AJCC cancer staging manual: continuing to build a bridge from a population-based to a more "personalized" approach to cancer staging. CA Cancer J Clin.

[3] Migden MR, Rischin D, Schmults CD (2018). PD-1 blockade with cemiplimab in advanced cutaneous squamous-cell carcinoma. N Engl J Med.

[4] Samaké M, Dembélé SB, Dianessy Y (2025). Squamous cell carcinoma of the scalp: a case report (Article in French). Health Sci Dis.

[5] Martorell-Calatayud A, Sanmartín Jimenez O, Cruz Mojarrieta J, Guillén Barona C (2013). Cutaneous squamous cell carcinoma: defining the high-risk variant. Actas Dermosifiliogr.

[6] van Dam V, Trinh XB, An B, Julien L (2021). Extra-anogenital giant cutaneous squamous cell carcinomas require multidisciplinary management. Cancer Treat Res Commun.

[7] Fitzpatrick TB (1988). The validity and practicality of sun-reactive skin types I through VI. Arch Dermatol.

[8] Wang K, Li Z, Chao SW, Wu XW (2022). Giant cutaneous squamous cell carcinoma of the popliteal fossa skin: a case report. World J Clin Cases.

[9] Gruber I, Koelbl O (2021). Dramatic radiotherapy response of a giant T4 cutaneous squamous cell carcinoma of the scalp with extensive bone destruction: a case report. J Med Case Rep.

[10] Mateuszczyk MK, Chlebicka I, Łyko M, Maj J, Szepietowski JC (2024). Extra-anogenital giant cutaneous squamous cell carcinomas. Life (Basel).

[11] Keohane SG, Botting J, Budny PG (2021). British Association of Dermatologists guidelines for the management of people with cutaneous squamous cell carcinoma 2020. Br J Dermatol.

[12] Barlow BR, Marshall RV, Wofford JD (2016). Giant cutaneous squamous cell carcinoma requiring emergent embolization. JAAD Case Rep.

[13] Trignano E, Tettamanzi M, Rampazzo S (2023). Squamous cell carcinoma of the scalp: a combination of different therapeutic strategies. Case Reports Plast Surg Hand Surg.

[14] Félix C, Cabrera F (2024). Giant cutaneous squamous cell carcinoma. J Clin Images Med Case Rep.

[15] López PA, Pedraza M, Moreno A (2019). Multidisciplinary management of giant cervicothoracic cutaneous squamous cell carcinoma. Int J Surg Case Rep.

